# The effects of three weight management methods on body composition and serum lipids of overweight and obese people

**DOI:** 10.3389/fnut.2022.1073576

**Published:** 2022-12-08

**Authors:** Jingjing Cai, Lin Shao, Shilong Zhao, Wen Liu, Peng Liu

**Affiliations:** ^1^Department of Nutrition, Peking University People’s Hospital, Beijing, China; ^2^Beijing Key Laboratory of Environmental Toxicology, School of Public Health, Capital Medical University, Beijing, China

**Keywords:** 5 + 2 IF, 5 + 2 intermittent fasting, body mass index (BMI), calorie-restricted diet (CRD), high protein diet (HPD), obesity, serum lipids

## Abstract

**Introduction:**

Obesity has become a major health problem worldwide. Diet management is an important means of weight loss. The aim of this study was to explore the improvement effect of a calorie-restricted diet (CRD), 5 + 2 intermittent fasting (5 + 2 IF), and high protein diet (HPD) on weight composition and serum lipid level of overweight and obese people in a short period (3 months).

**Methods:**

Fifty-three participants aged 18–60 years and with body mass index (BMI) ranging from 24 to 35 kg/m^2^ were screened for inclusion and exclusion criteria and were randomly divided into three groups, i.e., CRD, 5 + 2 IF, and HPD. Basic information, body composition, and venous blood samples were collected at baseline and after 3 months of intervention. Body composition was measured using a body composition analyzer (SK-V9), and serum lipids were assayed using the Roche Cobas e702 automatic biochemistry analysis system. The generalized estimating equation (GEE) was used to analyze and compare the repeated measurements of body composition and levels of serum lipids.

**Results:**

The results showed that total weight, BMI, body fat mass, muscle mass, visceral fat index, and waist and hip circumferences had significantly decreased in all three groups after 3 months of intervention, and the average weight loss was 3.3 ± 1.14, 4.12 ± 0.05, and 2.62 ± 0.13 kg in CRD, 5 + 2 IF, and HPD groups, respectively. The results of the GEE model indicated that compared with the CRD group, the 5 + 2 IF group showed a more significant decrease in weight (β = −0.272, *P* < 0.001), BMI (β = −0.091, *P* < 0.001), body fat mass (β = −0.172, *P* < 0.001), muscle (β = −0.043, *P* < 0.001), and visceral fat index (β = −0.019, *P* < 0.001), however, HPD has more advantages in visceral fat index loss (β = −0.011, *P* < 0.001) and lean body mass preserve (β = 0.229, *P* < 0.001).

**Conclusion:**

Our findings showed that the 5 + 2 IF may be more effective in reducing total weight and body fat, and HPD may be more helpful in preventing lean body mass loss during a short-term weight loss intervention.

## Introduction

Obesity is defined as “abnormal or excessive fat accumulation that may impair health,” which has become a major health problem worldwide and ranked as the sixth most common leading cause of death and disability in 2019 (1, 2). China has the highest number of overweight and obese people in the world; according to the Chinese criteria, about half of the adults are overweight or obese (3). Being overweight and obese are risk factors for many non-communicable diseases (NCDs), such as type 2 diabetes mellitus, cardiovascular disease, neurodegenerative diseases, and certain cancers (4). However, some studies have considered that the distribution of adipose tissue rather than the amount has shown a strong adverse association with the development of NCDs (5).

Comprehensive lifestyle interventions (CLIs) have been the foundation of the management of weight loss continuously, including the following three critical components: behavioral, dietary, physical activity change, and to produce a negative energy balance (6). Calorie-restricted diet (CRD) has recommended a daily caloric deficit of approximately 500 kcal (women: 1,200–1,500 kcal/day; men: 1,500–1,800 kcal/day) (7). Intermittent fasting (IF) is a more flexible calorie restriction strategy, which requests individuals to alternate between fasting and usual intake based on time, in which “fasting” days of 500–800 kcal intake (8). The efficiency of metabolic regulation and weight loss can be affected by circadian rhythms as energy homeostasis is maintained by the interaction of peripheral signals with the central nervous system (9). Besides, a high protein diet (HPD) might be a novel strategy for the weight loss of overweight and obesity. HPD adjusts the proportion of dietary protein in the energy supply of the macronutrients, which could promote satiety, and energy expenditure and in changing body composition in favor of lean mass (10). Changes in the macronutrient composition affect hormones, metabolic pathways, gene expression, and the composition and function of the gut microbiome which might impact fat storage (9). Although the above three weight management methods have significant weight loss effects, which can effectively lose weight in a short period and improve metabolic indicators, this needs to be confirmed by further research.

The present study reviewed the metabolic indicators of body weight, body composition, and serum lipids before and after 3 months of weight loss management in the participants who have participated in the CRD, 5 + 2 IF, and HPD in the weight management clinic, and we would like to explore the safety and effective weight loss management in the short term.

## Materials and methods

### Participants

All participants gave written informed consent to this study, and this study obtained a favorable opinion from the Human Ethics Committee of the Peking Union Medical College Hospital (2020PHD007-001). The participants were overweight or obese staff volunteers who participated in weight loss intervention at the nutrition and weight loss Clinic of Peking University People’s Hospital from August to September 2020. Inclusion criteria were 18–60 years of age and body mass index (BMI) ranged from 24 to 35 kg/m^2^. The exclusion criteria were (1) secondary obesity caused by endocrine or genetic, such as hypothalamic obesity, pituitary obesity, hypothyroidism obesity, obesity caused by Cushing’s syndrome, and hypogonadism obesity; (2) unable to consume test protein intake due to intolerances/dietary preferences/severe liver and kidney diseases; (3) those who are dieting or trying to lose weight recently; (4) pregnant or planning to become pregnant (within 3 months) or currently breastfeeding; and (5) metabolic health disturbance, malignancy, or other severe illnesses, such as diabetes, cancer, coronary heart disease, stroke, severe gastrointestinal diseases, or treatment that may interfere with study variable.

### Experimental design

Fifty-three participants underwent a 3-month intervention with three different weight management methods, and the sample size of this study is combined with the research demand. A computer-generated random number was used to randomly divide the patients into three groups, i.e., 18 people were on a CRD, 18 people were on a 5 + 2 intermittent fasting (5 + 2 IF), and 17 people were on an HPD. CRD is a dietary pattern that limits energy intake while ensuring basic nutritional needs. The energy supply ratio of macronutrients meets the requirements of a balanced diet. Based on satisfying the five nutrients of proteins, vitamins, minerals, dietary fibers, and water, moderately reduce the intake of fat and carbohydrates, and reduce the energy of normal diet by 30–50% of the low-energy dietary pattern; 5 + 2 IF, in which participants fasted for 2 days per week (either consecutively or non-consecutively) with total caloric intake 1/4 of the usual energy (approximately 500 kcal for women and 600 kcal for men) and 5 days of *ad libitum* eating; HPD recommended participants daily intake for protein at 1.5 g/(kg body weight daily), or 20∼30% of the total daily energy intake. In this study, participants in the HPD group consumed protein with 20∼25% of the total daily energy intake.

Researchers were professional nutritionists and dietitians. Participants were asked to follow up with the weight loss clinic once a week to strengthen the personalized diet plan, and nutrition education will be carried out online every 2 weeks. A group management approach to quality control of subjects’ implementation of weight management methods was through WeChat.

### Outcome measures

Researchers have collected basic information on participants at baseline and after 3 months of intervention, including gender, age, waist and hip circumferences, systolic pressure (SBP), and diastolic pressure (DBP). Body composition including height, weight, BMI (BMI was calculated as weight kg/height m^2^), body fat mass, muscle mass, and visceral fat index (visceral fat index = visceral fat area cm^2^/10 cm^2^) was measured and calculated using a body composition analyzer (SK-V9).

### Laboratory methods

Notably, 6 ml of venous blood samples for biochemical parameters were obtained in the morning after overnight fasting (more than 10 h) for analysis. Whole blood and plasma were collected in vacutainer tubes with anticoagulant Ethylenediaminetetraacetic acid (EDTA), and the relevant indicators were tested in a timely manner. Serum levels of total cholesterol (TC), triglyceride (TG), low-density lipoprotein cholesterol (LDL-C), and high-density lipoprotein cholesterol (HDL-C) were assayed using the Roche Cobas e702 automatic biochemistry analysis system. Serum levels of alanine transaminase (ALT), aspartate transaminase (AST), total bilirubin (TBIL), direct bilirubin (DBIL), urea, creatinine (CRE), and uric acid (UA) were assayed using the BECKMAN COULTER AU5800 automatic biochemistry analysis system, and Roche and BECKMAN kit for auto-analyzer were used.

### Statistics

Continuous variables are presented as mean ± SD or median (interquartile range), and categorical variables are presented as frequencies (percentages). One-way analysis of variance (ANOVA) or Kruskal–Wallis rank sum test for continuous variables and the chi-square test or Fisher exact test for categorical variables were run to determine whether there were differences between groups at baseline. The generalized estimating equation (GEE) was used to analyze and compare the repeated measurements of weight, BMI, body fat mass, muscle mass, visceral fat index, and serum lipids among the three groups at baseline and after 3 months of intervention. All the studies were collected by questionnaire and analyzed using SPSS (version 24.0), and GraphPad Prism 8.0 software was used to draw statistical figures. *P* < 0.05 was considered statistically significant.

## Results

### Characteristics of participants

A summary of the characteristics of 53 participants at baseline is shown in Table 1. There were 18 people in the CRD group, 18 people in the 5 + 2 IF group, and 17 people in the HPD group. At baseline, three groups were of balanced sex, age, height, ALT, AST, TBIL, DBIL, urea, CRE, UA, SBP, and DBP.

**TABLE 1:** Baseline characteristics of the study population.

Variables	CRD (*N* = 18)	5 + 2 IF (*N* = 18)	HPD (*N* = 17)	*P*-values
*N*, male (%)	4, 22.2%	1, 5.6%	6, 35.3%	0.094
Age, years	43.226 ± 10.474	45.333 ± 9.641	34.765 ± 8.074	0.68
Height, cm	164 ± 9.02	161.47 ± 5.9	165.94 ± 9.36	0.547
ALT, U/L	29.94 ± 35.45	32.56 ± 24.53	36.94 ± 31.52	0.59
AST, U/L	23.22 ± 11.12	27.56 ± 12.44	26.24 ± 13.39	0.392
TBIL, μmol/L	12.49 ± 4.24	15.32 ± 10.76	17.99 ± 11.9	0.74
DBIL, μmol/L	3.89 ± 1.16	4.74 ± 3.8	6.22 ± 6.25	0.899
Urea, mmol/L	4.28 ± 1.11	4.48 ± 1.08	4.1 ± 0.93	0.561
CRE, μmol/L	69.06 ± 13.73	69.11 ± 14.05	69 ± 14.26	0.999
UA, μmol/L	384.44 ± 82.57	362.06 ± 95.18	382.47 ± 92.7	0.715
SBP, mmHg	121.39 ± 13.26	123.61 ± 13.59	120 ± 13.11	0.722
DBP, mmHg	78.33 ± 8.40	78.61 ± 8.01	78.82 ± 7.81	0.984

5 + 2 IF, 5 + 2 intermittent fasting; ALT, alanine transaminase; AST, aspartate transaminase; CRD, calorie-restricted diet; CRE, creatinine; DBIL, direct bilirubin; DBP, diastolic pressure; HPD, high protein diet; SBP, systolic pressure; TBIL, total bilirubin; UA, uric acid. Data were expressed as % for categorical variables, using the chi-square test or Fisher’s exact test; mean ± SE for normally distributed variables, using Student’s *t*-test. Median (IQRs) for skewed distributions, using Kruskal–Wallis test.

### Body composition

**FIGURE 1:**
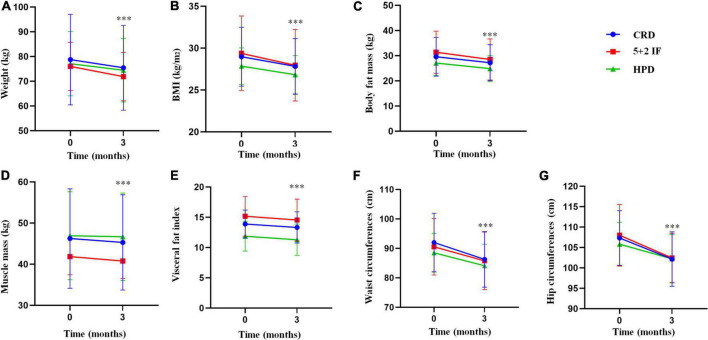
Results of body composition and body mass index (BMI) among the treatment and control groups. 5 + 2 IF, 5 + 2 intermittent fasting; BMI, body mass index; CRD, calorie-restricted diet; HPD, high protein diet; SE, standard error. The figure shows the level of body composition before and after three weight management methods by mean ± standard deviation [**(A)** weight; **(B)** BMI; **(C)** body fat mass; **(D)** muscle mass; **(E)** visceral fat index; **(F)** waist circumference; **(G)** hip circumference], ***P < 0.001.

As shown in Figure 1, there was no significant difference in weight, BMI, body fat mass, muscle mass, visceral fat index, waist circumference, and hip circumference of CRD, 5 + 2 IF, and HPD at baseline (*P* > 0.05). Among CRD, 5 + 2 IF, and HPD groups, after 3 months of intervention, the average weight loss was 3.3 ± 1.14, 4.12 ± 0.05, and 2.62 ± 0.13 kg, respectively; the average BMI reduction was 1.15 ± 0.194, 1.422 ± 0.19, and 1.024 ± 0.112, respectively; the body fat mass loss was 2.35 ± 0.56, 2.86 ± 0.2, and 2.23 ± 0.23 kg, respectively; the muscle mass reduction was 0.94 ± 0.52, 1.07 ± 0.17, and 0.25 ± 0.06 kg, respectively; the visceral fat index was decreased by 0.56 ± 0.27, 0.61 ± 0.16, and 0.59 ± 0.14, respectively; the waist circumference was decreased by 5.72 ± 0.47, 4.78 ± 0.18, and 4.41 ± 0.73 cm, respectively; the hip circumference was decreased by 5.16 ± 0.02, 5.56 ± 1.52, and 3.58 ± 0.57 cm, respectively.

Table 2 shows the results of the GEE model adjusting gender, age, group, time, and interaction factors of group and time. The results show that, compared with the baseline, the three weight management methods are (β = −1.1, *P* < 0.001), BMI (β = −0.383, *P* < 0.001), body fat mass (β = −0.738, *P* < 0.001), muscle mass (β = −0.313, *P* < 0.001), visceral fat index (β = −0.185, *P* < 0.001), waist circumference (β = −1.907, *P* < 0.001), and hip circumference (β = −1.722, *P* < 0.001). Compared with the CRD group, the 5 + 2 IF group showed a more significant decrease in weight (β = −0.272, *P* < 0.001), BMI (β = −0.091, *P* < 0.001), body fat mass (β = −0.172, *P* < 0.001), muscle (β = −0.043, *P* < 0.001), and visceral fat index (β = −0.019, *P* < 0.001), however, HPD has more advantages in visceral fat index loss (β = −0.011, *P* < 0.001) and lean body mass preserve (β = 0.229, *P* < 0.001).

**TABLE 2:** Adjusted model for body composition and body mass index (BMI) results at baseline and 3 months among three groups in a randomized, placebo-controlled trial.^a^

Body composition	Variables	β	SE	*P*-values^b^
Weight, kg	Time	−1.1	0.0144	<0.001
	CRD	Ref		
	5 + 2 IF	−0.575	4.4005	0.896
	HPD	−5.411	4.6898	0.249
	CRD × time	Ref		
	5 + 2 IF × time	−0.272	4.401	<0.001
	HPD × time	0.229	4.4689	<0.001
BMI, kg/m^2^	Time	−0.383	0.0038	<0.001
	CRD	Ref		
	5 + 2 IF	0.528	1.1645	0.65
	HPD	−1.715	1.2411	0.167
	CRD × time	Ref		
	5 + 2 IF × time	−0.091	0.0054	<0.001
	HPD × time	0.042	0.0055	<0.001
Body fat mass, kg	Time	−0.783	0.0077	<0.001
	CRD	Ref		
	5 + 2 IF	1.198	2.3517	0.611
	HPD	−2.469	2.5063	0.325
	CRD × time	Ref		
	5 + 2 IF × time	−0.172	0.0109	<0.001
	HPD × time	0.042	0.0111	<0.001
Muscle mass, kg	Time	−0.313	0.008	<0.001
	CRD	Ref		
	5 + 2 IF	−1.669	2.4412	0.494
	HPD	−2.968	2.6017	0.254
	CRD × time	Ref		
	5 + 2 IF × time	−0.043	0.0113	<0.001
	HPD × time	0.229	0.0115	<0.001
Visceral fat index	Time	−0.185	0.0022	<0.001
	CRD	Ref		
	5 + 2 IF	0.443	0.6748	0.511
	HPD	−0.58	0.7191	0.42
	CRD × time	Ref		
	5 + 2 IF × time	−0.019	0.0031	<0.001
	HPD × time	−0.011	0.0032	<0.001
Waist circumferences, cm	Time	−1.907	0.1664	<0.001
	CRD	Ref		
	5 + 2 IF	−0.365	2.9222	0.901
	HPD	−4.83	3.1121	0.121
	CRD × time	Ref		
	5 + 2 IF × time	0.315	0.2353	0.181
	HPD × time	0.437	0.2387	0.067
Hip circumferences, cm	Time	−1.722	0.2574	<0.001
	CRD	Ref		
	5 + 2 IF	1.046	2.1547	0.627
	HPD	−2.587	2.2894	0.259
	CRD × time	Ref		
	5 + 2 IF × time	−0.13	0.364	0.722
	HPD × time	0.526	0.3693	0.154

5 + 2 IF, 5 + 2 intermittent fasting; BMI, body mass index; CRD, calorie-restricted diet; HPD, high protein diet; SE, standard error.

^a^Generalized linear mixed model adjusted for the group, time, group × time, sex, year, β, SE, and *P*-values by the group is displayed.

^b^*P*-values are significant at *P* < 0.05.

### Serum lipids

The results of the model have an adjustment for gender, age, group, time, and interaction factors of the group, and time showed that TC, TG, HDL-C, LDL-C, and LDL-C/HDL-C did not differ among the three groups, and the specific results are shown in Table 3.

**TABLE 3:** Adjusted model for serum lipid results at baseline and 3 months among three groups in a randomized, placebo-controlled trial.^a^

Serum lipids	Variables	β	SE	*P*-values^b^
TC	Time	−0.054	0.0381	0.158
	CRD	Ref		
	IF	−0.103	0.2892	0.721
	HPD	−0.09	0.307	0.77
	CRD × time	Ref		
	IF × time	0.027	0.0538	0.615
	HPD × time	−0.014	0.0546	0.801
TG	Time	−0.092	0.0575	0.109
	CRD	Ref		
	IF	0.165	0.2344	0.481
	HPD	0.335	0.2466	0.175
	CRD × time	Ref		
	IF × time	0.078	0.0813	0.337
	HPD × time	−0.067	0.0825	0.415
HDL-C	Time	−0.006	0.0085	0.46
	CRD	Ref		
	IF	−0.137	0.0932	0.143
	HPD	0.03	0.0992	0.762
	CRD × time	Ref		
	IF × time	0.002	0.0121	0.89
	HPD × time	0.013	0.0122	0.304
LDL-C	Time	−0.041	0.0632	0.514
	CRD	Ref		
	IF	0.124	0.247	0.615
	HPD	−0.04	0.2595	0.877
	CRD × time	Ref		
	IF × time	0.018	0.0894	0.841
	HPD × time	−0.029	0.0907	0.748
LDL/HDL	Time	−0.04	0.0504	0.422
	CRD	Ref		
	IF	0.217	0.2541	0.394
	HPD	−0.114	0.2685	0.672
	CRD × time	Ref		
	IF × time	0.043	0.0712	0.544
	HPD × time	−0.045	0.0723	0.533

5 + 2 IF, 5 + 2 intermittent fasting; CRD, calorie-restricted diet; HDL-C, high density lipoprotein cholesterol; HPD, high protein diet; LDL-C, low density lipoprotein cholesterol; SE, standard error; TC, total cholesterol; TG, triglyceride.

^a^Generalized linear mixed model adjusted for the group, time, group × time, sex, year, β, SE, and *P*-values by the group is displayed.

^b^*P*-values are significant at *P* < 0.05.

## Discussion

Our findings found that all three weight management diets had significant effects on weight loss, BMI, total fat, visceral fat index, and waist and hip circumferences. Compared with the other two weight loss diets, 5 + 2 IF is more effective in reducing weight, BMI, total fat content, and visceral fat; an HPD is more helpful in preventing muscle mass loss while reducing weight.

Meta-analysis suggests that IF is associated with statistically significant weight loss of more than 5% in adults with overweight or obese, and participants were found to be significantly decreased by 1.67 kg (95% CI, −2.79 to −0.55) following 3 months of the 5 + 2 diet (11–13). A study on overweight and obese women (*n* = 115) aged 20–69 years with a family history of breast cancer found that the IF diet is superior to the CRD diet with respect to the improvements in insulin sensitivity and the loss of body fat (14). Michelle et al.’s study on 107 patients with overweight and obese found that the average weight loss of the 3-month CRD diet group was 4.1 kg, and the average weight loss of the IF was 3.0 kg (15). In this research, the average weight loss of the 5 + 2 IF group was 4.12 kg after 3 months, accounting for 5.3% of the baseline weight, which was consistent with the research before. In addition, our study shows that the 5 + 2 IF group is superior to the CRD group and the HPD group in reducing BMI, body fat mass, visceral fat index, and waist and hip circumferences. A meta-analysis suggested that IF diets were associated with higher weight loss and fat mass decrease compared with a continuous energy restriction (CER) regimen (16), which shows that the 5 + 2 IF has greater advantages in short-term (3 months) weight loss.

A total of 8 weeks of randomized clinical trials (RCTs) suggested that HPD appeared to be more beneficial than a low glycemic index (GI) diet, as indicated by the greater fat mass loss and preservation of muscle mass (17). Our study suggests that the average weight loss of the HPD group was 2.62 kg, accounting for 3.4% of the baseline weight. The HPD group was slightly inferior to the CRD group and the 5 + 2 IF group in terms of total weight loss, BMI, and body fat loss, but better than the CRD group in terms of visceral fat index reduction. In addition, the HPD group had the least reduction in muscle mass, which was more conducive to reducing the loss of lean weight than the CRD and the 5 + 2 IF group. A recent meta-analysis suggested that supplying whey protein to overweight and obese individuals decreased body fat mass, preserved lean body mass, and improved metabolic waist circumference (10, 18, 19). This may indicate that protein plays a key role in preventing muscle loss during weight management.

An umbrella review of 11 meta-analyses of RCTs indicated that modified alternate-day fasting (MADF) was also found to be associated with the improvement of several cardiometabolic risk factors including LDL-C, TC, TG, and blood pressure (11), and this indicates that MADF may have the effect of improving serum lipids, although our study has not found the improvement effect of 5 + 2 IF on blood lipid. An RCT found that compared with obese patients who added maltodextrin to their diet, obese patients who added whey protein-lipid profiles in plasma have improved (20), and this shows that whey protein may play a role in improving serum lipids. Our research has not found that HPD can reduce blood lipids. We speculate that it might have normal blood lipids and blood pressure at baseline, and it was not surprising that most metabolic risk indicators were unchanged.

Our study has compared the effects of CRD, 5 + 2 IF, and HPD on weight loss, BMI, body fat mass, muscle mass, visceral fat index, waist circumference, hip circumference, and serum lipids within 3 months, and this study provides a reference for short-term weight loss and body fat mass reduction, which is innovative and practical. Weight loss can effectively improve metabolic indicators and prevent the occurrence and development of diseases related to overweight and obesity, such as diabetes and cardiovascular and cerebrovascular diseases. This study provides three methods that can effectively reduce body weight and improve lipid metabolism and distribution with certain clinical significance. However, this study also has some limitations. Due to the short-term of the study, the effect of three weight management methods on improving serum lipids has not been found, which needs to be confirmed by further longer and larger sample size studies. Besides, we lack using a tool to assess dietary intake as well; in addition, we will increase observation time points in future research and analyze inflammation, glucose metabolism, and other indicators to further explore the effects of three weight loss methods on metabolism and explore the mechanism.

## Conclusion

Three ways of weight management have significant reduction effects on weight loss, body fat mass, visceral fat index, and waist and hip circumferences. The 5 + 2 IF has the most significant effect on total weight loss and body and visceral fat reduction, and HPD has more advantages in preserving muscle mass.

## Data availability statement

The raw data supporting the conclusions of this article will be made available by the authors, without undue reservation.

## Ethics statement

The studies involving human participants were reviewed and approved by the Human Ethics Committee of the Peking Union Medical College Hospital. The patients/participants provided their written informed consent to participate in this study.

## Author contributions

PL conceived and designed the study. JC and WL performed the analyses and wrote the manuscript. LS and SZ collected and analyzed the data. All authors read and approved the final manuscript.
