# Corrigendum: The effects of three weight management methods on body composition and serum lipids of overweight and obese people

**DOI:** 10.3389/fnut.2023.1139098

**Published:** 2023-02-01

**Authors:** Jingjing Cai, Lin Shao, Shilong Zhao, Wen Liu, Peng Liu

**Affiliations:** ^1^Department of Nutrition, Peking University People's Hospital, Beijing, China; ^2^Beijing Key Laboratory of Environmental Toxicology, School of Public Health, Capital Medical University, Beijing, China

**Keywords:** 5 + 2 IF, 5 + 2 intermittent fasting, body mass index (BMI), calorie-restricted diet (CRD), high protein diet (HPD), obesity, serum lipids

In the published article, there was an error in the author list. Lin Shao and Shilong Zhao are erroneously listed as sharing first authorship.

The corrected author list appears below.

Jingjing Cai^1^, Lin Shao^1^, Shilong Zhao^1^, Wen Liu^2*^ and Peng Liu^1*^

The authors apologize for this error and state that this does not change the scientific conclusions of the article in any way. The original article has been updated.

